# A Conceptual Framework for Psychosocial Support of Orphaned and Vulnerable Children in the Vhembe District, South Africa

**DOI:** 10.1002/puh2.70045

**Published:** 2025-03-11

**Authors:** Livhuwani Precious Matshepete, Lufuno Makhado, Ntsieni Stella Mashau

**Affiliations:** ^1^ Department of Public Health, Faculty of Health Sciences University of Venda Thohoyandou South Africa; ^2^ Office of the Deputy Dean Research and Postgraduate Studies, Faculty of Health Sciences University of Venda Thohoyandou South Africa

**Keywords:** development, OVC, programme, psychosocial, support

## Abstract

**Background:**

The lack of a conceptual framework (CF) that can be utilized to manage the psychosocial well‐being of orphans and vulnerable children present a challenge in the Vhembe district, South Africa.

**Aim:**

This article sought to provide a CF for psychosocial support of OVC in the Vhembe district, South Africa using Practice‐Oriented Theory and Donabedian's structure Outcome Model features.

**Method:**

The study incorporated a four‐phased research strategy using a multiphase mixed methods approach. An exploratory, descriptive design was used for the study. The practice‐oriented theory of Dickoff et al. and Donabedian's SPO model provided a starting point in the ultimate development of the framework. Data were collected from three sources, orphans and vulnerable children *n* = 34, to explore and describe the experiences conducted with community‐based workers working with orphaned and vulnerable children within the Vhembe district to explore possible approaches for psychosocial support towards orphans and vulnerable children *n* = 4, CBOS, and social workers *n* = 10 working with OVC in the Vhembe district to explore the possible approaches for psychosocial support towards OVC until data saturation.

**Results:**

The study revealed that OVC faces many challenges including loss/absence of parents, living arrangements, deprivation and neglect, abuse and alienation. Challenges exist and this was confirmed by the qualitative findings with community‐based workers and social workers, including lack of resources, lack of proper training, lack of funding and poor relationship between stakeholders and the absence of the CF that guide the provision of psychosocial support to OVC.

**Conclusion:**

The study findings were conceptualized to describe and develop a framework for psychosocial support of OVC in order to improve the psychosocial well‐being of OVC.

## Graphical abstract

The study revealed that OVC faces many challenges, including loss/absence of parents, living arrangements, deprivation and neglect, abuse and alienation. Challenges exist and this was confirmed by the qualitative findings with community‐based workers and social workers, including lack of resources, lack of proper training, lack of funding and poor relationship between stakeholders and the absence of the conceptual framework that guide the provision of psychosocial support to OVC.

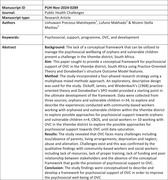

## 1 Introduction and Background

Orphan hood and the vulnerability of children as a result of parents' death remain a serious global concern. Human immunodeficiency virus (HIV) has been reported as the leading contributory factor of orphan hood and vulnerability of children [1]. Due to the prevalence of HIV in Africa and other parts of the world, millions of people have died from the virus, leaving millions of children without parents and exposed to abuse [2]. Orphanhood and vulnerability of children because of parental death related to the epidemic and other circumstances have exposed these children to a range of challenges [3]. According to Bimha et al. [3], HIV and AIDS have left lots of children receiving insufficient health care, insufficient food, and inadequate education, thereby increasing their vulnerability to all sorts of abuse and poor mental health. These tough experiences demonstrate that psychosocial support to orphans and vulnerable children is a necessity.

The current statistics show that Eastern and Southern Africa continue to be the two regions most severely affected by HIV, with approximately 55% of all people (20.6 million) and two‐thirds of children living with the virus [1]. There were 2.8 million children who were reported to be orphan in South Africa [4]. This constitutes 14% of all children living without parents in the country. South Africa has recorded 1728,000 (9%) paternal orphans, 530,000 (3%) maternal orphans, and 505,000 (2%) of double orphans [4].

Nevertheless, South Africa has made the continuing determination to lessen the spread of the Virus and was one of the four Southern African countries that have achieved 73% of viral suspension in 2020 [1]. The South African Government, through the Department of Social Development (DSD), released a draft for OVC care in the form of a policy framework for OVC [5]; the next year, it handed out a national action plan for OVC. Both the framework and the action plan offer a clear path for addressing the social impact of HIV and AIDS and for providing services to OVC, with priority on family and community care and with institutional care viewed as a last resort. The six main approaches of the action plan consist of

- strengthening the capacity of families to care for OVC;
- mobilizing community‐based responses for care, support and protection of OVC;
- ensuring that legislation, policy, and programmes are in place to protect the most vulnerable children;
- ensuring access to essential services for OVC;
- increasing awareness and advocacy regarding OVC issues;
- engaging the business community to support OVC actively [5].

Most programmes for OVC focus much on meeting the physical needs of children [6]. The importance of psychosocial support services studies cannot be overrated. The psychosocial effect of HIV on children is often overlooked. The loss of a parent is a traumatic and stressful experience. There is a lack of support in helping the OVC to cope with the trauma related to witnessing the deaths of family members [7]. The additional problem of caring for terminally ill relatives may send children into shock, leaving many of them with unanswered questions about their own future.

The objective of this study was to develop a conceptual framework (CF) for psychosocial support of orphaned and vulnerable children to improve the psychosocial well‐being of the OVC. In this article, the practice‐oriented theory of Dickoff et al. [8] and Donabedian's structure, process, and outcome model formed the bases for the development of developing a CF.

The study's aim was to conduct meta‐inferences and an interpretation of the overall study findings to develop a CF.

## 2 Research Methods and Design

A qualitative multiphase mixed method was chosen using an exploratory descriptive design. To obtain an in‐depth understanding of the impact of psychosocial support to OVC in the Vhembe district of South Africa, an exploratory design was used because the researcher wanted to explore the experiences of OVC, the possible approaches for psychosocial support towards OVC by community workers as well as social workers. The researchers also wanted the participants of the study to contribute to the development of new knowledge in the area of psychosocial support of OVC [9]. A descriptive design helps the researchers to describe the views of OVC regarding their experiences and also helped researchers in grouping the information, formulating themes and subtheme that guided the CF [9]

The CF was developed on the basis of the imperial results of the three qualitative studies. The practice‐oriented theory of Dickoff et al. [8] and Donabedian's structure process and outcome models were concurrently used to classify and categorize the characteristics, activities and functions of a CF. There is a mutual relationship between Dickcoff's practice‐oriented theory and Donabedian's SPO model. Therefore, the two provided a starting point for the development of the CF.

The typical characteristics, activities, and functions were outlined and described through the process of abstraction, starting from the concrete level of experience to the higher level of abstraction to determine an ideal framework [10].

The results of the abstraction purely identified the characteristics of the development of a CF. As a result, this study goes through the selection of characteristics by further examining and describing the relationship among the typical characteristics, activities and functions of a CF [10].

The exhaustiveness and mutual exclusiveness were used as criteria to select the most appropriate characteristics best describing the phenomenon. Once more, further refinement was done to remove overlapping activities [10].

**Table:** 

Research designs and methods for the development of a conceptual framework					
	Design	Population	Sampling	Sample size	Context
Phase 1	Exploratory descriptive	OVC	Purposive sampling	34	Rural
Phase 2	Exploratory descriptive	Community‐based workers	Purposive sampling	4 CBOs	Rural
Phase 3	Exploratory descriptive	Social workers	Purposive sampling	10 social workers	Rural
Phase 4	Conceptual framework	OVC, Community‐based workers and social workers	Non‐probability purposive sampling	34 OVC, 4 CBOs, 10 social workers	Rural

### 2.1 Sample and Study Setting

A non‐probability purposive sampling was used to select *n* = 34 OVC, *n* = 4CBOs and *n* = 10 social workers within the four municipalities in the Vhembe district. This method makes sure that participants were selected on the basis of their relevant experiences and met particular standards, permitting the researcher to collect the most relevant data probable [11]. Limpopo province has four districts, namely, Capricorn, Sekhukhune, Waterberg, and Vhembe. The researchers purposively selected Vhembe district based on high prevalence of orphans and vulnerable children. Vhembe District has recorded the highest number of maternal (12,575) and paternal (29,746) orphans [4]. The researchers included all the municipalities within the Vhembe District to get an equal sample representation.

### 2.2 Data Collection

Data were collected from 34 OVC to using face‐to face interviews to explore and describe the experience of OVC. Furthermore, four focus group discussions with *n* = 4CBOs, which lasted for 40–60 min, were used to collect data from CBWs. Face‐to‐face interviews were conducted with *n* = 10 social workers, which lasted for about 40–60 min. Data saturation was reached within this number. The researcher noticed an advantages in using face‐to‐face interviews and focus group discussions as they allowed for first‐hand information and observation, and easy with data recording [12]. The focus group discussions produced data representing different opinions, perceptions and views from community‐based workers regarding the possible approaches for psychosocial support for OVC [12]. An in‐depth semi‐structured interview was used for this study because the researcher wanted to understand participant's lived experiences, feelings, perceptions or situations from their point of view in their own words as described by Fouche et al. [12]. Furthermore, the researchers were able to engage with participants to explore participants’ experiences from their point of view in their own words about psychosocial support of OVC. In this article, we are reporting the integrated findings of three empirical studies to develop a CF for psychosocial support of OVC in the Vhembe district of South Africa.

### 2.3 Data Analysis

Data analysis was done in accordance with Creswell and Creswell's [13] suggestions and Tesch's open code data analysis guide. The procedure included transcription of the original text word for word, individual readings of the transcript to improve understanding, grouping the data into themes and arranging these topics into columns. Next, emerging themes were categorized and connected to relevant data points. Tables arranged according to topics, categories and subcategories were used to methodically convey the analytical results.

### 2.4 Trustworthiness

The trustworthiness of the study was heightened using a multiphase mixed methods design; close attention was paid to elements that were more significant to the study by adhering to the research methods and honestly reporting data. The researchers also provided a thick description of the research findings and an audit trail kept. Data were also organized in categories and themes. The researcher also kept all audio‐recorded data and notes safe for future reference.

## 3 Results and Discussion

The practice‐oriented theory of Dickoff et al. [8] and the SPO model of Donabedian [14] served as the foundation for the development of the CF. Donabedian's [14] SPO and Dickoff et al.’s [8] practice‐oriented theory both helped the researcher identify and synthesize the various elements that resulted in the developing phase 1, 2 and 3 outcomes as well as, ultimately, a CF.

Because of its characteristics, which include the structure, process, and outcome, Donabedian's SPO model was applied. The practice‐oriented theory of Dickoff et al. [8] has the following characteristics: the agent, recipients, context, procedure, dynamic, and endpoint. In order to attain the intended result, an SPO model includes the agent, recipients and stakeholders in the structural process [14]. The dynamic must be studied separately, but the terminus must be included into the result because they have the same meaning [8, 14]. Here is how the CF is created and explained.

### 3.1 The Structure

According to Donabedian‘s [14] structure process outcome model, the structure refers to the place in which it is necessary to have structural resources, required equipment and personnel to provide required psychosocial supports. According to Dickoff et al. [8], an agent is a person or thing in the structure that is in charge of carrying out an activity, and it is embedded within the structure. In this study, the agents included the social workers and community‐based workers. It is evident from the study findings that they lack a CF to guide the provision of a psychosocial support programme that strives to achieve the intended goal of improved psychosocial well of OVC. In most cases, it does not address the gaps affecting the psychosocial support programme. Ahmad [15] argues that transparent and accountable governance structures are essential for the efficient and ethical management of resources. These structures ensure rigorous financial oversight, transparent decision‐making processes and robust child protection measures, safeguarding the well‐being of children in care

According to Dickoff et al. [8], the recipient is referred to the person or thing that getting services from the agent and it is also incorporated in the structure. In this framework, recipients were OVC from Vhembe district receiving psychosocial support from social workers and community‐based workers. The results of the study qualitative phase study conducted with OVC about the experiences of OVC identified the following experiences, namely, loss/absence of parents, living arrangements, deprivation and neglect, abuse and alienation. The OVC reported the following during the interview:

I lost my father in 2016, and I lost my mother in 2020 during COVID‐19, and it was painful for me since my mother was the only person who was taking care of me. I miss her every day and my life is difficult without her (Female, participant number 22, age 14).

**Another participant expressed that**:

The treatment is very bad because I am the only one doing home chores, once I arrived home from here, I am not allowed to play with other children, do not have time to do my homework (Female, participant 16, age 14**)**.

In support of this study, a study conducted by Alem [16] reported that when parents die, children do not only miss their physical presence but also many positive things they provided them with when they were alive, such as love, guidance, care and protection. In addition to that Kiambi and Mugambi [17] stated that OVC faces various forms of abuse and exploitation: physical violence, defilement, sexual exploitation, child labour and child marriage, whereas more and more people take to the streets to protect themselves. The study found that OVC are experiencing lots of challenges, which influence the need for psychosocial support. Macleod [18] further indicated that the need for care and affection comes at the third level of Maslow's hierarchy of needs. This shows that for a child to be a completely functioning psychologically and emotionally being, they must have people who care for them and love them. Several studies have also reported that OVC normally experience neglect, abandonment or abuse [19, 20]. It is evident from the study results that the agent should provide comprehensive quality psychosocial support to the OVC.

### 3.2 Process

The process follows the structure, and it is defined as the guiding principles [14]. According to Dickoff et al. [8], the process refers to the rule, technique, protocol and routine leading the actions to achieve the terminus. The process that was outlined in this framework was the process that must be carried out when providing psychosocial support to OVC. The study's results in two qualitative phases (focus group with community‐based workers and semi‐structured face‐to face interview with social workers) revealed that community‐based workers and social workers identified several suggestions to improve psychosocial support to orphans and vulnerable children. The suggestions from the studies finding include the following: Provision of quality training, and provision of adequate resources. The social workers expressed that

Another thing needed is continuous training for us social workers because the department is not training us in the form of workshops on the new developments regarding rendering psychosocial support or on working with orphans and vulnerable children in general (Participant 6).

In support of this, a study conducted by Gumede [21], reports that ongoing training and skill development is necessary for community‐based workers and social workers to continue rendering their valuable services to OVC:

I think if our department can provide us with necessary resources to do our jobs like cars, telephone, computer, printer, and all other office stationaries, rendering psychosocial support to the OVC would improve (Participant 1).

The findings of this study revealed that social workers suggested the involvement of various stakeholders in policy development to improve the psychosocial support of orphans and vulnerable children. The social workers indicated that

The guardians that are raising these children and social workers need to be included when developing those policies to voice their opinions because the guardians are the ones staying with those children, and they know what those children need, and we as social workers are dealing with them daily (Participant 6).

And

I think policies must be done by the people working with these children who know what they are going through because sometimes, as social workers, we fail to assist the child just because there are some of the things that I might think of doing to help the child and only to find that the policy does not allow me to do (Participant 5).

In support of this study findings, Green and Brown [22] posit that community engagement initiatives that involve local stakeholders in the upbringing of orphans have been shown to create a more supportive and inclusive environment. Moreover, this holistic approach not only supports sustainable practices within the institution but also prepares children to become environmentally conscious citizens in the long term, contributing to broader sustainability efforts in their communities [23]. Involving children in decision‐making processes within orphanages, as advocated by UNICEF [24], is crucial. Empowering children to participate in decisions that affect their lives fosters a sense of ownership and belonging, leading to improved developmental outcomes. Furthermore, research highlights that CBOs with transparent governance structures and active stakeholder involvement experience higher levels of trust and stability [15].

The community‐based workers in a focus group reported lack of funding, lack of proper training, lack of infrastructure, poor relationship with other social service professionals, as the main challenges affecting the provision of effective psychosocial support of OVC. The community‐based workers expressed the following:

The main challenge that our organization is facing now is that we are not funded, we are sustaining ourselves through donations and is really affecting our services (Female participant 4 from CBO A).

Another participant expressed that:

We are not trained properly in the aspects of psychosocial support, the only training that we are getting is from DSD in which we will be grouped and capacitated about working with OVC, maybe for one day and is not enough and we end up not rendering our services effectively (Female participant 6 from CBO D).

Another male participant 2 from CBO B reported that “We do not have a proper infrastructure to cater for all our OVC and during rainy season we are not operating hence we are using a shack. This is really affecting our service delivery”.

In addition to that another participant verbalized that

Our relationship with social workers is not that good because most of the time when we refer a case to them, they do not take our case serious (Female participant 1 from CBO C).

In support of this study's findings, a study conducted by Ahmad [15] reported that resource limitations hinder the adoption of essential technologies, whereas insufficient funding poses obstacles to sustainable practices [15]. Moreover, inadequate awareness among staff and management necessitates training programs to enhance understanding and implementation [15]. In addition, Nkirote [25] emphasizes the need to build long‐term infrastructure and developed training and compensation to enhance this workforce potential. Murerwa [26] reported that lack of good professional relationships makes it difficult for CBWs and social workers to render effective and quality services. It is evident from the study that quality training, mentoring; supports and compliance with policies and guidelines are the guiding principles that facilitate the achievement of psychosocial support programme outcomes. Once more, monitoring, reporting and evaluation facilitate the identification of gaps, signs of danger and success to come to the terminus. Nonetheless, there is a need for dynamic power sources to sustain operations in between. According to the CF, the source of power enables an agent to follow the recommended procedures (rules, regulations, and guidelines) in order to increase the success of the outcomes.

### 3.3 Outcome (Terminus)

**FIGURE 1:**
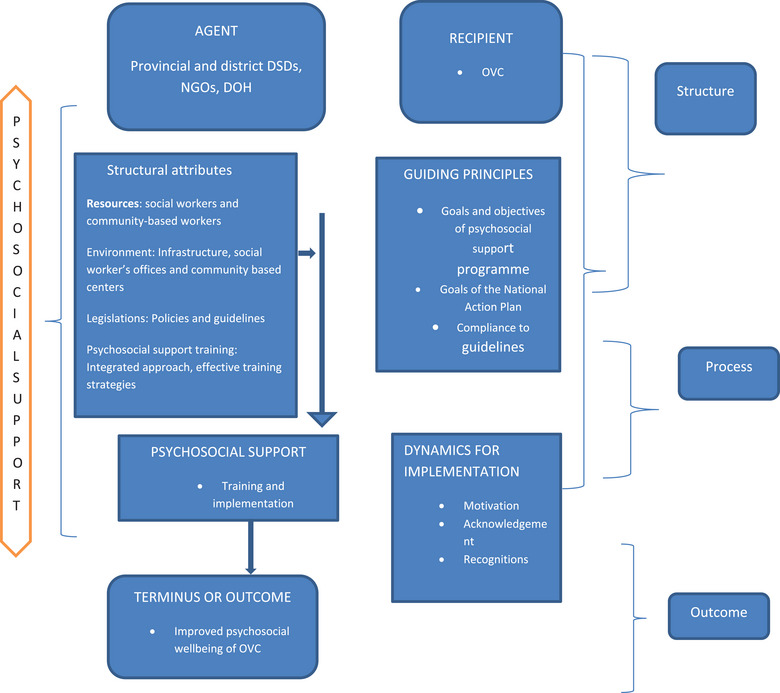
A conceptual framework for a psychosocial support programme to support OVC.

Terminus refers to the outcomes or result of the activity [8]. Donabedian [14] describes a terminus as the end product or outcomes of the structure and process. It is clear from the study findings that the outcomes of an effective psychosocial support programme facilitate the production of improved psychosocial well‐being of the OVC. Dickoff's six survey list and Donabedian's SPO were categorized and classified with the characteristics and activities from the study findings to develop a CF that can facilitate and influence the development of a psychosocial support programme to support the OVC in Vhembe district thus improve outcome as presented in Figure 1

### 3.4 Context

According to Dickoff et al. [8], the context refers to resources, activities and environment that enable or facilitate implementation. It is evident from the study that a combination of organizational resources and a conducive, safe and comfortable environment within the DSD can facilitate implementation. The availability of adequate, independent, experienced, skilled community‐based workers and social workers with a positive attitude towards OVC and psychosocial support programmes for OVC strengthens the implementation. Moreover, providing psychosocial support pre‐service training to community‐based and social workers can facilitate the implementation. The availability of good communication and relationship skills, including compliance with OVC policies and guidelines, facilitates implementation.

## 4 Conclusion

The psychosocial support programme has a significant impact on the well‐being of the OVC as it resulted in the improved psychosocial well‐being of OVC in the Vhembe district. However, the quality regarding psychosocial support programme is still a challenge even though social workers and community‐based workers are providing it. It is evident from the study results that various factors such as infrastructures, lack of continuous trainings, lack of funding and client influence the implementation of a psychosocial support programme. The developed CF has the potential to strengthen the implementation of the psychosocial support programme, thereby enhancing the psychosocial well‐being of OVC. The CF also indicated the importance of allowing various stakeholders to work together when providing psychosocial support services to OVC, thus promoting effective provision of psychosocial support services to OVC.

## 5 Recommendations

On the basis of the research findings, the study recommends that

- The CF be adopted for enhancing the psychosocial well‐being of OVC.
- The DSD should provide adequate resources to social workers and community‐based workers so that they could be able to provide psychosocial support services to OVC effectively.
- The DSD should improve social workers’ morale by improving their conditions of services.

## 6 Limitations of the Study

The study was limited to one district in the Limpopo province only. Regardless of this limitation, the study findings are important as no such CF exists in the province.

## 7 Practical Implications of the Study

With reference to the study findings, the CF developed could help the implementation of psychosocial support programme in the Vhembe district, thus improving the psychosocial well‐being of OVC.

## Author Contributions

**Livhuwani Precious Matshepete**: investigation; resources; conceptualization; methodology; validation; formal analysis; data curation; writing – review and editing; visualization. **Lufuno Makhado**: writing – original draft; writing – review and editing; data curation; supervision; formal analysis. **Ntsieni Stella Mashau**: formal analysis; visualization; supervision.

## Ethics Statement

The study received approval from the Research Ethics and Social Sciences Committee of the university of Venda. The University of Venda's Ethical clearance number for the study is FHS/23/PH/05/0706. The Limpopo Provincial DSD was also approached for permission and it was granted. The data collected were audio‐recorded after permission was granted, and this was kept in a safe and locked. Potential harm to participants was avoided at all costs. Confidentiality and anonymity in all procedures were upheld for ethical conduct [12].

## Consent

Voluntary, written consent was obtained from participants, who were informed about their rights to withdraw from the study at any time.

## Conflicts of Interest

The authors declare no conflicts of interest.

## Data Availability

The data that support the findings of this study are available on request from the corresponding author. The data are not publicly available due to privacy or ethical restrictions.
